# Smoking and microbiome in oral, airway, gut and some systemic diseases

**DOI:** 10.1186/s12967-019-1971-7

**Published:** 2019-07-15

**Authors:** Chunrong Huang, Guochao Shi

**Affiliations:** 1 Department of Pulmonary and Critical Care Medicine, Ruijin Hospital, Shanghai Jiao Tong University School of Medicine, 197, Rui Jin Er Road, Shanghai, 200025 People’s Republic of China; 2 Institute of Respiratory Diseases, Shanghai Jiao Tong University School of Medicine, 197, Rui Jin Er Road, Shanghai, 200025 People’s Republic of China

**Keywords:** Microbiome, Oral, Lung, Gut, Disease

## Abstract

The human microbiome harbors a diverse array of microbes which establishes a mutually beneficial relation with the host in healthy conditions, however, the dynamic homeostasis is influenced by both host and environmental factors. Smoking contributes to modifications of the oral, lung and gut microbiome, leading to various diseases, such as periodontitis, asthma, chronic obstructive pulmonary disease, Crohn’s disease, ulcerative colitis and cancers. However, the exact causal relationship between smoking and microbiome alteration remains to be further explored.

## Background

Approximately 2 billion people worldwide use tobacco products, mostly in the form of cigarettes, with tobacco smoking-related diseases resulting in at least 4 million global deaths per year [1]. Dramatic rise of diseases associated with cigarette smoke or tobacco use, including cardiovascular disease, chronic obstructive pulmonary diseases (COPD), Crohn’s disease, and various forms of cancer [2], implying the potential detrimental role of smoking in occurrence of human diseases. Emerging evidence suggests that environmental factors play an influential role in shaping human-associated microbial communities and immune responses. Either active smoking or exposure to secondhand smoke is associated with colonization by potentially pathogenic bacteria [3–5]. Yet, in an era where microbes not only cause acute infectious illnesses but also are increasingly being recognized as etiologic agents or risk factors for chronic diseases including cancers [6–8] and neurologic disorders [9, 10], it is important to have a profound understanding of the effect of smoking on microbiome in diseases.

The microbiome refers to a community of microbes residing in a defined environment, comprising of bacteria, viruses, fungi, and protozoa, together with their genes and genomes in a given locus. The gastrointestinal microbiome is the most complex echo-system of 10–100 trillion microorganisms, in which the amount of bacteria was the most, in the next place was that of fungi and virus [11]. The oral communities come as the second in the human body [12]. With the initiation of the Human Microbiome Project in 2007, the use of culture-independent methods allied with next generation DNA sequencing methods to identify the composition of the human microbiome, is providing a far deeper analysis than hitherto possible [13], including 16S ribosomalRNA (rRNA) sequencing, metagenomic sequencing, and microbial metatranscriptomics [14]. The once called sterile lung based on conventional culture methods was unraveled to contain variable microbiomes depending on health and specific disease states [15, 16]. The human microbiome has the stability and resilience to restore themselves after perturbation maintains homeostasis in health, but its composition is susceptible to many factors such as antibiotics, diet, alcohol, and smoking [17] (Table 1). It has become clear that the microbiome is not a passive victim in many pathological processes, but its modification often play a contributive or causative role in pathophysiological processes [18]. Thus far, most studies have described the microbial composition of healthy or diseased organs, and smoking associated alterations of microbiome in different sites were demonstrated in a variety of diseases (Table 2). In this review, we will summarize the current understanding of the impact of smoking on microbiome and its involvement in various diseases, and thereby highlighting important research questions that require further investigation.

**Table 1:** Alterations of microbiome in healthy smokers

	Reference	Origin	Sample	Enriched microbes	Depleted microbes
Oral	[37]	Human	Subgingival plaque	Species: *Fusobacterium nucleatum*, *F. naviforme*, *Filifactor alocis*, *Dialister microaerophilus*, *Desulfobulbus* sp. clone R004, *Megasphaera sueciensis*, *M. geminatus*, *M. elsdenii*, *M. micronuciformis*, *Acinetobacter johnsonii*, *A. guillouiae*, *A. schindleri*, *A. baumannii*, *A. haemolyticus*, *Pseudomonas pseudoalcaligenes*, *Pseudoramibacter alactolyticus*	Species: *Streptococcus sanguinis*, *S. parasanguinis*, *S. oralis*, *Granulicatella elegans*, *G. adiacens*, *Actinomyces viscosus*, *A. israelii*, *A. dentalis*, *Neisseria subflava*, *Hemophilus parainfluenzae*
	[191]	Human	Oral wash samples	Genera: *Atopobium*, *Bifidobacterium*, *Lactobacillus*, *Streptococcus*	
	[192]	Human	Mouth wash sample	Phylum: *Spirochaetes*, *Synergistetes* and *Tenericute*s, *Bacteroidetes* and *Actinobacteria* Genera: *Treponema*, TG5 and *Mycoplasma*, *Megasphaera*, *Dialister*, *Paludibacter*, *Porphyromonas*, *Prevotella*, *Atopobium*	Phylum: *Proteobacteria*, *Fusobacteria*, SR1, GN02 and *Cyanobacteria* Genera: *Neisseria*, *Eikenella*, *Aggregatibacter*, *Actinobacillus*, *Haemophilus*, *Lautropia*, *Fusobacterium*, *Leptotrichia*
Airway	[193]	Human	Nasopharyngeal swab, oropharyngeal swabs	Oropharynx Genera: *Megasphaera*, *Veillonella* spp. Nasopharynx Genera: *Eggerthella*, *Erysipelotrichaceae* I.S., *Dorea*, *Anaerovorax*, *Eubacterium* spp.	Oropharynx Genera: *Capnocytophaga*, *Fusobacterium*, *Neisseria* spp. Nasopharynx Genera: *Shigella* spp.
	[194]	Mice	Lung sample	Genera: *Trichococcus*, *Escherichia*–*Shigella*, *Oxalobacteraceae*	Genera: *Oceanospirillales*, *Lactobacillus*, *Lactobacillaceae*, *Enterobacter*, *Acidimicrobiales*_norank, *Caulobacteraceae*_ *Phyllobacteriaceae*_uncultured, *Raoultella*, *Caulobacteraceae*_unclassified
	[88]	Human	BALF	Virome: *Prevotella*, *Xanthomonas*, *Actinomyces*, *Aeromonas*, *Capnocytophaga*, *Haemophilus* and *Rhodoferax* phages	Virome: *Lactobacillus*, *Gardnerella* phages, *Enhydrobacter*, *Enterobacter*, *Holospora*, *Morganella*, *Enhydrobacter*, and *Spiroplasma* phages
Gut	[124]	Rat	Caecal contents	Not reported	Genera: *Bifidobacterium* sp.
	[125]	Mice	Caecal contents	Genera: *Clostridium* sp.	Genera: *Lactococcus* sp., *Ruminococcus* sp., *Enterobacteriaceae* sp. and segmented filamentous bacteria
	[175]	Mice	Colonic sample	Genera: *Lachnospiraceae* sp.	Not reported

**Table 2:** Influence of smoking on the microbiome in some diseases

Diseases	Reference	Origin	Sample	Enriched microbes	Depleted microbes
Periodontitis	[60]	Human	Subgingival plaque sample	Genera: *Fusobacterium*, *Fretibacterium*, *Streptococcus*, *Veillonella*, *Corynebacterium*, TM7, *Filifactor*	Genera: *Prevotella*, *Campylobacter*, *Aggregatibacter*, *Veillonellaceae* GQ422718, *Haemophilus*, *Prevotellaceae*
Asthma	[104]	Human	Subgingival plaque sample	Genera: *Fusobacterium*, *Prevotella* and *Selenomonas*	Not reported
Crohn’s disease	[128]	Human	Subgingival plaque sample	Genera: *Anaeroglobus*, *Bulleidia*, *Corynebacterium*, *Granulicatella*	Genera: *Veillonella*, TM7

### Possible mechanism of the impact of smoking on microbiome

Massive studies demonstrated the adverse health impacts of tobacco on systemic pathophysiologic changes that can lead to disease, were associated with the chemicals, heavy metals, particulate matter and other constituents in tobacco [19–27]. However, a paucity of studies investigated the microbes in tobacco recent years, and this may be incriminated as causative factors in smoking-associated diseases. Before advances in DNA sequencing technology, the golden standard of identification of microbes-culture method, was used to identify the *Pantoea agglomerans*, *Acinetobacter calcoaceticus*, and specific *Pseudomonadaceae* species such as *P. fluorescens* and *Stenotrophomonas maltophilia* in fresh tobacco leaves or other species in single tobacco flakes or fine tobacco particles [24, 26]. With the advent of high-throughput sequencing technology, a 16S rRNA-based taxonomic microarray and cloning and sequencing were utilized to identify a variety of uncultured species. Cigarettes made in the European Union contained 15 different classes of bacteria. Sapkota et al. revealed extensive bacterial diversity in cigarettes, ranging from soil microorganisms and commensals to potential human pathogens, including *Acinetobacter*, *Bacillus*, *Burkholderia*, *Clostridium*, *Klebsiella*, and *Pseudomonas aeruginosa*. Many of the detected organisms are capable of causing pneumonia, bacteremias, foodborne illnesses, meningitis, endocarditis, and urinary tract infections [28]. Therefore, mechanism that may lead to different bacteria profiles among smokers may be due to exposure to bacteria in cigarettes, leading to bacterial acquisition and colonization.

Another possibility for the mechanism through which current smokers may have different bacteria community may be related to impaired antimicrobial defenses due to the immunosuppressive nature of tobacco. Tobacco smoking has been observed to affect the peripheral immune system on several levels, including a decrease in the activity of natural killer cells, increase in white blood cell counts, and a higher susceptibility to infection [29]. Smoking increases the number of macrophages, neutrophils, eosinophils, and mast cells, decreases the number of airway dendritic cells, and alters macrophage and neutrophil function [30, 31]. Expanding macrophages and neutrophils demonstrated impaired phagocytic functions to the efficient clearance of bacteria or pathogen, as evidenced by reduced bacterial-stimulated production of superoxide and surface receptor expression, (e.g. TLR2) which is important for the recognition and response to gram-positive bacteria [32, 33]. Therefore, smoking related immunosuppression could permit novel bacteria colonization.

It is also possible that metabolic advantages of biofilm formation and increased adherence to the epithelium are conferred to certain taxa expansion in a smoky environment. Exposure to cigarette smoke could increase biofilm formation by specific bacteria [34, 35]. Biofilm is a self-generated polymer matrix that insulates the pneumococcus and other microbial pathogens from host defense and antibiotics, promoting bacterial persistence [36]. Mutepe et al. found that increased biofilm formation of *Streptococcus pneumoniae* and inactivation of pneumolysin induced by exposure to cigarette smoke condensate are likely to favor microbial colonization and persistence, both being essential precursors of pneumococcal disease [35]. Similarly, in another study, observations of increased biofilm formation of *Staphylococcus aureus* and human cell adherence in the presence of cigarette smoke (CS) indicate the role of bioactive effects of CS on resident microbiota in the pathogenesis of respiratory infection in CS-exposed humans [34]. These findings suggest that cigarette smoke may promote colonization and persistence of specific bacterial taxa in the human body through the biofilm formation, contributing to infections in different parts of the body.

“Microenvironment” may also be relevant regarding the influence of smoking on particular members of the microbiota, such as oxygen, pH, and acid production. Oxygen tension is an important promoter of the changes in bacterial community, with microaerophilic and anaerobic bacteria able to predominate due to lower oxygenation [37, 38]. Shanahan et al. demonstrated a reduction of the relative abundance *Prevotella* and *Neisseria* spp. and an increased relative abundance of *Firmicutes*, principally *Streptococcus* spp., and *Veillonella* spp., along with the genus *Rothia* (*Actinobacteria*) in the upper GI tract from current smokers, compared with that from persons who have never smoked [39]. The differences observed in *Neisseria*, *Streptococcus*, and *Rothia* spp. in current smokers indicated the implication of changes in oxygen tension. In that study, alterations in duodenal bicarbonate secretion [40] and lower pH [41] in smokers may also provide selective pressure on the growth of *Neisseria*, which is one of the capnophiles and sensitive to acid conditions [42], whereas *Streptococcus* and *Rothia* spp. are acidogenic and acid tolerant.

**Fig. 1:**
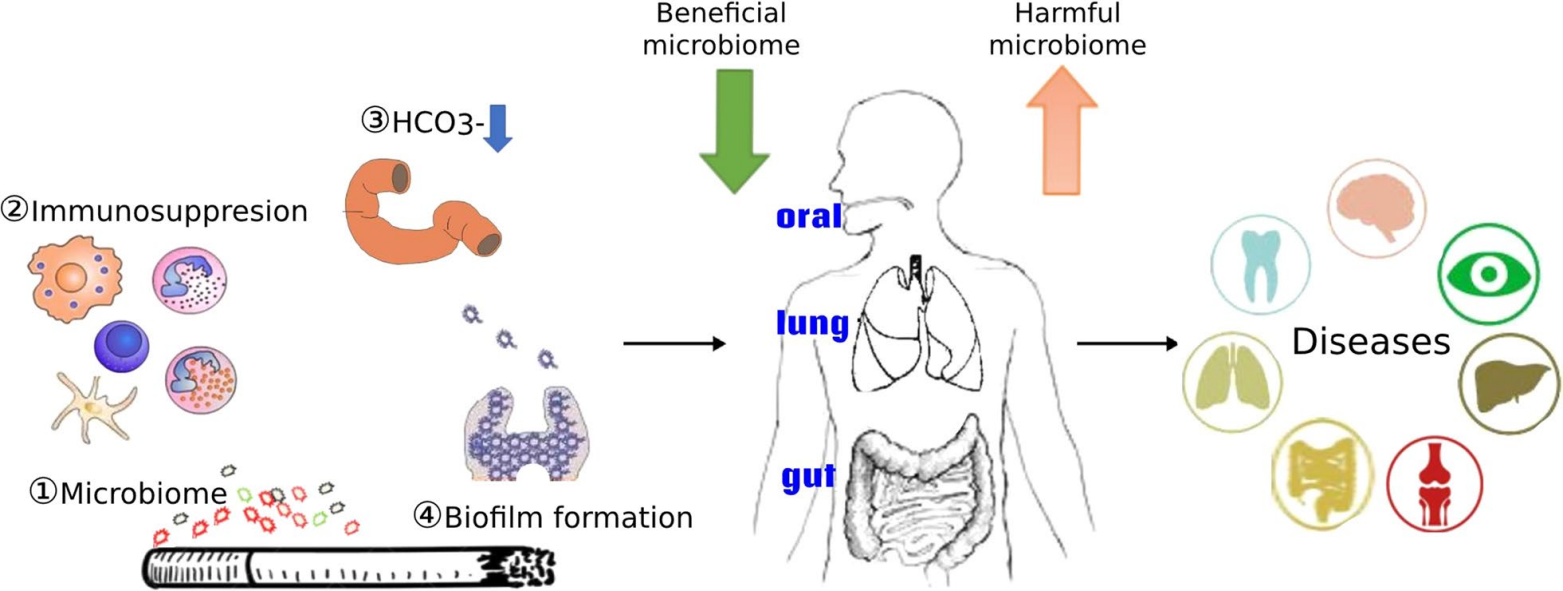
Schematic summary illustrating the possible mechanisms of smoking-induced dysbiosis of microbiome and its possible role in different diseases

According to above discussion, we could reach the conclusion of mechanisms of cigarette smoking to influence the microbiome via changes to immune homeostasis, biofilm formation, oxygen tension, or through direct contact with microbes it contained, and these mechanisms may be involved in the occurence of various diseases (Fig. 1).

### Smoking and oral microbiome in diseases

The oral microbiome, comprising more than 2000 bacterial species [43], plays an important role in the maintenance of oral health [44]. Dysbiosis of oral microbiota has been associated locally with periodontal, respiratory, cardiovascular and systemic cancers, including head and neck cancer [45], pancreatic cancer [46], and esophageal cancer [47], yet regarding factors that influence the oral microbiome composition are poorly understood. Smoking is a major environmental factor that influences orodental pathophysiology [48]. Toxic components and bacteria in cigarette impact oral bacteria directly or indirectly through immunosuppression, oxygen deprivation, biofilm formation, or other potential mechanisms [49], leading to loss of beneficial oral species and pathogen colonization, ultimately to disease [50]. Despite of different sampling sites or laboratory methodologies, numerous studies have shown predominant or inhibited genera in oral from smokers compared with non-smokers. Culture results of smokers showed less numerous *Neisseria* species or *Branhamella* [51, 52]. Due to limitations on bacterial profiling of traditional method, recently, sequence analysis of bacterial 16S rRNA-encoding genes was performed to identify the different mouth communities between nonsmokers and smokers in species such as *Porphyromonas*, *Neisseria*, and *Gemella* [53]. Mason et al. revealed the microbial profiles of subgingival plaque samples from 200 systemically and periodontally healthy smokers and never-smokers were different at all taxonomic levels, and principal coordinate analysis revealed distinct clustering of the microbial communities based on smoking status. Smokers demonstrated a highly diverse, pathogen-rich, commensal-poor, anaerobic microbiome that is more closely aligned with a disease-associated community in clinically healthy individuals, suggesting that it creates an at-risk-for-harm environment that is primed for a future ecological catastrophe [37].

#### Periodontitis

Evidences indicate that periodontitis was associated with smoking and complex microbial communities in the subgingival sulcus [54–56], and cigarette smokers were found to have a statistically significant higher risk of severe periodontitis than non-tobacco users [57]. More and more researchers focused on the associations between smoking and sub-gingival bacterial species in the pathogenesis of periodontitis. Smoking-associated periodontitis is less diverse and distinct from that of non-smokers. Shchipkova et al. explored that the microbial profile of smokers with moderate to severe chronic periodontitis and demonstrated significant differences in the prevalence and abundance of disease-associated and health-compatible organisms, with greater abundance of *Parvimonas*, *Fusobacterium*, *Campylobacter*, *Bacteroides*, and *Treponema* and lower levels of *Veillonella*, *Neisseria*, and *Streptococcus* [58]. The differences existed in the composition of the subgingival microbiome between smoker and non-smoker patients with chronic moderate periodontitis were also elucidated in other studies [59–61]. In addition, smokers are at high risk for other oral diseases, such as peri-implant mucositis and peri-implantitis [62, 63]. Tsigarida et al. demonstrated smoking shapes the peri-implant microbiome of peri-implant biofilm samples from patients with peri-implant health, peri-implant mucositis, and peri-implantitis [63], paralleled with studies depicting that the underlying mechanism is through depleting commensals from this niche and favoring colonization of pathogens [63].

#### Infective endocarditis

In recent years, significant associations have been elucidated between periodontitis and other systemic diseases [64, 65], and the bacterial flora of the mouth entering the bloodstream may potentially be involved in the pathogenesis of invasive infections such as infective endocarditis, and the bacterial flora of the mouth entering the bloodstream may potentially be involved in the pathogenesis of invasive infections such as infective endocarditis [66]. Oral bacteria of endocarditis patients have been reported to be shown different community compositions from that of healthy individuals [67, 68]. *Staphylococcus aureus*, viri-dans *Streptococci* and *Enterococcus* spp. are the most common pathogens identified [69]. *Gemella sanguine*, *Streptococcus tigurinus*, *L. goodfellowi* were also found to be the cause of infective endocarditis [70–72]. However, there was barely study about the effect of smoking on infective endocarditis regarding oral microbiome. Biofilm formation, complex mechanisms with other bacteria might play a crucial role in the occurrence of invasive infections [71]. Indeed, endocarditis is also considered an example of a biofilm-mediated disease [73]. Given the importance of biofilm formation for adhesion in the oral cavity [70], and the fact that cigarette smoke increased biofilm formation by specific bacteria and promoted colonization [34], it would be reasonable to suspect that oral microbiome might be the missed connectivity between smoking and infective endocarditis. Further studies on whether smoking could increase the incidence of infectious endocarditis through altering oral micro-organisms remains to be elucidated.

#### Other

In addition to local health, oral microbiome also plays an important role in other systemic diseases, including HIV infection, gastrointestinal cancer, even immune diseases. HIV infection has been associated with dysbiosis of oral microbiome, with increased levels of pathogenic bacteria and fungi [74, 75], HIV-infected smokers showed rich abundance of specific bacterial taxa compared to infected non-smokers, including *Granulicatella*, *Lactobacillus*, *Veillonella*, *Enhydrobacter*, *Streptococcaceae* and *Comamonadaceae*, moreover, abundance of the fungal genus *Candida* was also increased in HIV-infected smokers [76]. Oral microbiome was also corroborated to be associated etiologically with gastrointestinal cancer in virtue of composition concordance among sites within the oral cavity and gut, and anatomical acquirement of gut microbiome from mouth. In colorectal cancer participants, current smoking was associated with a 33% decrease in relative counts of *Betaproteobacteria* (primarily *Neisseria*) and 23% increase in relative abundance of *Veillonellaceae* family [77]. These data indicated that community composition of oral microbiome may be associated with numerous diseases such as periodontitis, cancer, and diabetes, however, it remains to elucidate the causative relationship between a specific bacterium and the disease, and future work may also wish to consider this potential association.

### Smoking and airway microbiome

Because of the presence of a sparse microbiome, especially in healthy conditions, traditional standard microbiological culture-based methods can hardly detect microbes in healthy individuals, so the lung has historically been presumed as sterile. Over the past several years, culture-independent molecular methods springing up, lung microbial communities in healthy individuals showed a phenotype predominant by *Proteobacteria*, *Firmicutes* and *Bacteroidetes*, as evidenced by bronchoalveolar lavage samples of healthy adults, bacterial communities vary with different airways in terms of different airway microarchitecture, and documented changes in the lung microbiome in several lung diseases have been uncovered [78, 79].

Smoking, cigarette smoke exposure, tobacco smoke or pollutants in the air contact directly with the airway, through the way to the lungs, causing a variety of airway diseases, such as COPD, asthma, cystic fibrosis and lung cancer. In recent years, the effect of smoking on microbiome of lower respiratory tract attracted increasing attention. Mammen and Aethi proposed a revised “Vicious Circle”, suggesting that insults such as tobacco smoke impairs innate immune defenses, causing variations in the abundance, taxonomic composition and phylogenetic diversity of the lung microbiome. This, in turn, leads to maladaptive inflammatory responses, further impairment of lung defenses and further dysbiosis of the lung microbiome, setting up the vicious circle with its attendant consequences [80].

#### COPD

COPD is a chronic airway inflammatory disease that can be prevented and treated lung disease, characterized by a largely irreversible chronic obstruction of airflow. The course of the disease is featured and frequently aggravated by intermittent exacerbations, acute changes in the airway microbiome, for example by introduction of a new strain of a respiratory pathogen, lead to larger inflammatory responses, which present clinically as exacerbations of COPD [80], so changes in the composition and activity of the microbiome may be implicated in their appearances. Pragman et al., once described the lung microbiome in moderate and severe COPD patients, with the former dominated by *Actinobacteria* and *Proteobacteria*, the later by *Actinobacteria* and *Firmicutes*, and they also found a trend without significant difference that severe subjects contained more *Firmicutes* and less *Actinobacteria* and *Proteobacteria* than the moderate subjects [81]. However, in another cohort study, in both stable and exacerbated samples, the most prevalent phyla were *Proteobacteria*, *Firmicutes* and *Actinobacteria*, the most prevalent genera were *Streptococcus* and *Haemophilus*, exceeding half of the abundance of present bacterial microbiome [82]. Although these researchers also found no significant differences in bronchial microbiome between stability and exacerbation using 16S rRNA sequencing and shotgun metagenomic sequencing, functional metabolic capabilities showed significant changes in several pathways, indicating specific changes in the lung microbiome in the progression of COPD [82].

Smoking is the principal cause or initiating factor responsible for the development of the disease in COPD patients. It alters host–microorganism interaction dynamics in the airways, contributing to COPD [83]. The influence of cigarette smoke on the microbiome and the role of the microbiome in COPD are relatively new field with limited data. Erb-Downward et al. showed the diversity of bacterial communities in bronchoalveolar lavage fluid (BALF) from healthy smokers was similar to that from healthy never-smokers, and COPD patients, however, further results from obtained lung tissues of COPD patients unraveled the heterogeneity and diversity in the bacterial microbiota across different regions of the abnormal lung [79], suggesting specific changes in lung microbiome resulted from smoking in COPD patients participate in the occurrence of COPD or exacerbation. Respiratory tract infections, either viral or bacterial, are major causes of acute exacerbation of COPD (AECOPD) [84]. Cigarette smoke exposure is a well-known risk factor for important bacterial and viral infections in the respiratory tract [85]. In the lung ecosystem, virus also plays a pivotal roles in lung diseases, for example, phages could lead to immune-mediated microbial competition [86], opportunistic infection [87], therefore, alterations of lung viral communities could change the bacteriome leading to dysbiosis and disease progression in individuals (e.g., COPD). Gregory et al. performed the first study of the effects of smoking on the lung DNA virome, lung viromes profiles were statistically indistinguishable across smokers and nonsmokers, and viral diversity was significantly lower in the lungs of healthy smokers [88]. Statistical analyses revealed that changes in viral communities correlate most with changes in levels of arachidonic acid and IL-8, both potentially relevant for COPD pathogenesis [88]. These data imply the potential role of changes in viral communities induced by smoking in the development of COPD. Although the role of smoking on microbiome in COPD needs to be further investigated, early studies have suggested an association between lung microbiota and the clinical outcomes of disease.

#### Asthma

Asthma is a chronic airway inflammatory disease, characterized by reversible airway obstruction, chronic airway inflammation, and airway hyper-responsiveness [89–91]. There are an emerging number of studies shown the correlations between airway microbiome and the incidence, severity or reactivity of corticosteroid medications of asthma [92–95]. Asthmatic patients harbored higher abundance of *Proteobacteria* and lower *Bacteroidetes* phylum compared to healthy control as evidenced from samples of bronchial epithelial brushings [96], and the sputum microbiota in severe asthma patients differs from healthy controls and non-severe asthmatics, with a significant correlation between *Streptococcus* spp. and eosinophilia in severe asthma patients [94]. Airway colonization with distinct specific bacteria were also associated with the severity of airways obstruction, neutrophilic airway inflammation [97] and corticosteroid resistance in asthma [98]. While the bacterial communities in airway inflammatory disease have been extensively studied, fungal microbiota is still poorly characterized. The analysis of induced sputum revealed that 90 fungal species were more abundant in asthmatics [99], of which members of genera *Aspergillus* and *Penicillium* were significantly associated with impaired post-bronchodilator expiratory volume in 1 s in asthmatics [100]. Severe asthmatics were characterized by enrichment of *Aspergillus*; the relative abundance of *Aspergillus* increased approximately 15-fold compared to mild asthmatics [101]. However, whether these organisms are cause or result of the pathophysiology or medications in asthma remains to be determined.

As the main source of indoor air pollution, tobacco smoking, mostly in the form of cigarette smoking, is an important environmental factor influencing the outcomes of asthma. Smoking (including active and passive smoking) can not only cause frequent attacks of asthma, lead to rapid decline of lung function in asthmatic patients, but also reduce the therapeutic effect of glucocorticoid in asthmatic patients, making the condition of asthmatic patients difficult to control [102]. Microbial colonization of the lower airway may be shaped by smoking in asthma, accordingly, some of the increased risk and severity of pulmonary disorders in tobacco smokers with asthma [103] could still be mediated through smoking-induced changes in the lung microbiome. Simpson et al. extracted DNA from induced sputum in asthma patients and profiled microbial communities using 16S rRNA pyrosequencing, the results showed ex-smokers have a higher prevalence of phylum *Fusobacteria*, the phyla *Firmicutes* and *Bacteroidetes*, and a lower abundance of bacteria from phylum *Proteobacteria* compared with never-smokers. They also revealed an association between smoking and increased diversity of bacteria [104]. However, another study failed to demonstrate the association, because of no difference in bacterial dominance from induced sputum in asthma patients between ex-smokers and non-smokers, this lack of difference may be explained by the small sample size and different samples in that study [97]. More studies examining effect of smoking on airway microbiome in individuals with asthma are warranted.

#### Cystic fibrosis

Cystic fibrosis (CF) is a multi-organ disease with variable clinical characteristics, with pulmonary manifestations (e.g. bronchiectasis and chronic infection) being the prominent feature [105, 106]. Progressive lung disease driven by microbial colonization and inflammation remains the leading cause of morbidity and mortality in CF patients [106]. The lung environment in cystic fibrosis patients, characterized by depletion of the airway surface liquid layer leading to impediment of mucociliary clearance, is ideal for microbial colonization [107]. Culture-independent molecular methods allowed emerging threads of the features of the respiratory microbiome in CF to be gradually discovered in recent years. The lung microbiota fluctuated in a chronological way and associated with clinical states. In CF patients aged < 2 years, nontraditional taxa (e.g. *Streptococcus*, *Prevotella* and *Veillonella*) predominated, these species shifted to traditional CF taxa (*Pseudomonas*, *Staphylococcus*, *Haemophilus*, *Stenotrophomonas* and *Burkholderia*) [108] and remained relatively stable in older children (> 6 years) and adults, especially in clinical stability [109–111]. With improvement of biological diagnosis, fungal and viral colonization in CF moved into the focus. *Candida albicans* and *Aspergillus fumigatus* are commonly detected in CF sputum cultures and have also been associated with acute pulmonary exacerbations [112], and the number of fungal species detected in sputum fluctuated over time [113]. As for respiratory “virome” in CF, precious few data reported distinct phage communities in CF compared with non-CF patients [114].

Mutations of cystic fibrosis transmembrane conductance regulator (CFTR), an epithelial anion channel, are predominant in the cause of CF [115]. Smoking is one of the most important adverse factors affecting respiratory health. Recent researches pay close attention to the issue related to the impact of smoking in the pathology of CF and clinical outcomes. Campbell et al. [116] found tobacco smoke in patients with CF exhibited poor clinical status, including reduced lung function, and a higher number of pulmonary-related hospitalizations. Some other studies focus on the variations of genes within the context of tobacco smoking and detected the associations between smoke exposure and CTFR dysfunction [117, 118], which could in turn lead to deleterious effects on airway surface liquid secretion, enhanced mucus expression, reduced mucociliary clearance, chronic bacterial infection, and excess inflammation [119], rendering the main morbidity and mortality in CF patients because of the fact that lung inflammation and chronic respiratory infections alone account for nearly 95% of the morbidity and mortality in patients with CF [120]. In addition, smoking is known to stimulate mucosal linings and increase sputum production in the respiratory tract, raising the possibility of bacterial infections. Yet, there is no evidence to investigate the microbiome (e.g. bacteria) in CF patients in the context of smoking. Both the effect of smoking on microbiome in CF patients and the possible causal relationship between smoking-induced variations of microbiome, possibly through CTFR dysfunction, and the clinical manifestations of CF, could not be proved. We may assume the possible mechanism of detrimental effect of smoking on CF may be because the alterations in microbiome due to airway surface liquid secretion, enhanced mucus expression, reduced mucociliary clearance induced by loss of CTFR functions.

### Smoking and gut microbiome

The gastrointestinal microbiome is a complex echosystem of 10–100 trillion microorganisms composed of bacteria, virus and fungal species, that develops immediately after birth depending on multiple factors, and fluctuates or changes resulted from affection of a number of factors such as age, drugs (especially antibiotics), diet, alcohol, and smoking throughout the whole time growing up [11, 17]. In virtue of the most extensively focus on microbiota colonizing the intestinal tract, it has become clear that in healthy individuals, the microbiome is inclined to remain rather stable, with *Bacteroides*, *Faecalibacterium*, and *Bifidobacterium* being the most prevalent genera [121], and disturbance of the microbial equilibrium is associated with a variety of local and systemic diseases [17, 18].

Smoking prevalence is the leading cause responsible for developing Crohn’s disease (CD), colonic carcinoma, and systemic disease [122, 123]. The vulnerability of intestinal microbiome provokes interest in microbiome alterations in smoking environment. In animal models, cigarette smoke decreases organic acids levels and population of bifidobacterium in the caecum of rats [124]. Side-stream smoking increased the abundance of *Clostridium* and decreased the amount of *Lactoccoci*, *Ruminococcus*, *Enterobacteriaceae* and segmented filamentous bacteria (SFB) in the cecal microflora [125]. In human studies, smoking increased the probability of developing *Clostridium difficile* infection [126]. Current smokers displayed increased *Bacteroidetes* and decreased *Firmicutes* and *Proteobacteria* in gut microbiota composition community compared with never smokers [127]. Another study also revealed healthy smokers harbour higher *Bacteroides*–*Prevotella* (34.8%) than nonsmokers (24.1%) [128]. In addition, smoking status is also connected with variations in gut microbiome, as reported by Biedermann et al. that healthy individuals undergoing smoking cessation increased *Firmicutes* and *Actinobacteria*, while decreasing *Bacteroidetes* and *Proteobacteria* [129]. Many researchers investigated the role of smoking in gut microbiome in inflammatory bowel disease, we will discuss the effects of smoking on gut microbiome in several diseases.

#### Crohn’s disease

Crohn’s disease (CD) is a type of inflammatory bowel disease that mainly affect gastrointestinal tract, characterized by chronic inflammation due to defective mucosal barrier and greater intestinal permeability [130]. While the cause of CD is multifactorial, a combination of environmental, immune, and bacterial factors in genetically susceptible individuals [131–133]. Microbial dysbiosis is thought to be associated with either development or exacerbation of underlying Crohn’s disease [134]. CD patients have a relevant dysbiosis of the gastrointestinal microbiome, including reduction of normal commensal phyla (*Bacteroidetes* and *Firmicutes*), increase of pathogenic organisms (*E. coli*, *Campylobacter* species, and *Mycobacterium* species) [135], and a greater number of mucosal surface-associated bacteria with higher adherence and invasion compared with healthy control subjects [136]. Researchers also found CD patients possess a reduction in *Roseburia* spp., *Clostridium* and *Bacteroides* species, known to be producers of butyrate, which is fundamental to intestinal cell homeostasis and mucosal barrier integrity [137, 138]. These studies have shed light on the possible causative role of the dysbiosis of gut microbiota in CD.

Smoking is the best studied environmental risk factor for CD, exerting detrimental effects on mucosal barrier and greater intestinal permeability and disease susceptibility [139]. Accumulating data investigated the association between smoking and imbalance of intestinal microbiome [139]. Accordingly, several studies indicated that intestinal microbes could be an important link between smoking and CD [139]. Benjamin et al. found greater abundance of *Bacteroides*–*Prevotella* in smoking patients with CD compared with nonsmokers through fluorescent in situ hybridization using probes targeting 16S rRNA of bacteria [128]. In another study, Opstelten reported a reduced microbial gene richness and taxonomic diversity in smoking patients with CD, they further demonstrated a statistically significant reduction in specific genera *Collinsella*, *Enterorhabdus*, and *Gordonibacter* [140], which can produce urolithins with anti-inflammatory properties [141]. *Faecalibacterium* has immune-regulatory function to reduce IL-12 expression in peripheral blood mononuclear cell (PBMC) in vitro and increase IL-10 release, lower ileal mucosal *Faecalibacterium prausnitzii* is correlated with greater risk of recurrence following surgical resection in CD patients [142]. Murugananthan et al. revealed a reduction in the number of *Faecalibacterium prausnitzii* in inflamed mucosal tissue from smokers with active CD compared with non-smokers, the risk of post-operative CD recurrence may be predetermined at a pre-operative stage due to dysbiosis. These observed features of reduced gut microbiota may explain the persistent intestinal inflammation in CD patients in smoky environment, highlighting the possible role of microbes interacting with smoking and CD (or mediating the adverse effects of smoking in CD). However, the mechanisms through which smoking caused alterations in microbiota are unclear.

#### Ulcerative colitis

Ulcerative colitis (UC) is another type of chronic relapsing inflammatory disorder confined to the colorectal region and to the mucosal layer of the gastrointestinal tract. Previous studies have demonstrated aberrant microbiota deviations from gut homeostasis in UC patients, as evidenced by a low taxonomic diversity, decreases in *Firmicutes* and increases in *Proteobacteria* in UC gut microbiomes [143–145]. Proportions of *Fusobacteriaceae* family increased, *Bifidobacteria* and members of the *Faecalibacterium* taxon appeared to be compromised in gut microbiota of UC patients [146, 147], and further study suggested the reduced abundance of *Bifidobacteria* as a microbial biomarker to identify the intestinal dysbiosis triggering UC [147].

The understanding of pathogenesis and etiology of UC is still out of reach. It was presumed that genetically susceptible individuals of UC or patients with UC exhibited aberrant mucosal immune response against their gut microbiota [148, 149], which could result in productions of pro-inflammatory cytokines responsible for abnormal inflammation reaction in the digestive tract [150]. Environmental factors were deemed to trigger the onset and cause flares of inflammatory bowel disease. Smoking is among the most widely studied factors described in UC. The contradictory results of reverse associations between smoking and the natural history of UC has long been the topic of great interest. Some studies showed current smokers with UC are more likely to show milder disease, fewer hospitalizations, and reduced need for corticosteroid and immunosuppressant therapy compared with non-smokers [151]. Others did not find the protective role [152, 153]. Li et al. [154] suggested cigarette smoking could change the composition of intestinal microbiota, modulate mucus production and inhibit repairing of the gastrointestinal tract. Nevertheless, the main components of cigarette smoking has inflammatory-regulatory properties, for instance, Heme oxygenase-1 (HO-1) and carbon monoxide (CO) play a role in modulating cytokine expression and macrophage bactericidal activity, regulating intestinal homeostasis and mucosal immune responses to the enteric microbiota [155, 156], both of which were involved in the possible pathological process in UC. The exact mechanisms through which smoking is associated with alterations in microbiota are unclear. As yet, there is no data exploring the effect of smoking on gut microbiota in patients with UC. Smokers may have behavioral characteristics, such as diet, that predispose to a luminal and mucosal dysbiosis [157]. However, it is also possible that smoking has a direct influence on the microbiota, and this deserved to be mentioned in future research hotspots.

#### Colorectal cancer

Colorectal cancer (CRC) is considered a major public healthy issue, with approximately 700,000 deaths reported annually worldwide [158], ranking only second to lung cancer [159]. Chronic inflammation is a well-established factor associated with cancer onset, progression through mucosal disruption and the excess of reactive oxygen species (ROS) [160, 161]. At present, many scholars believe that the pathogenesis of CRC may be associated with the participation of intestinal microorganisms, which started the damage mechanisms of intestinal mucosal immune response, thus leading to immune response and inflammation [162]. The correlation between gut microbiome and the initiation of cancer can be dated from last century when people observed the potential etiology of bacteria Streptococcus bovis in the occurrence of CRC [163]. In recent years, the disturbance of gut microbiome was further pushed to a high new climax, as distinct gut microbiome composition detected in CRC patients.

The abundance of several microbes, such as *Streptococcus gallolyticus* [164], *Fusobacterium*, [165], *B. fragilis* [166], *Escherichia*–*Shigella*, *Peptostreptococcus* ten [167] were observed to be enriched in CRC patients versus control groups, while genera such as *Bacteroides*, *Roseburia* [164] and *Pseudomonas* [167] were significantly depleted in CRC patients. Moreover, the barely studied fields gut virome and mycobiome correlated with CRC were also reported when Nakatsu et al. identified a set of discriminatory virome signatures (e.g. Orthobunyavirus, Tunalikevirus, Phikzlikevirus, Betabaculovirus, Sp6likevirus, Sfi21dtunalikevirus, Punalikevirus, Lambdalikevirus, C2likevirus, and Mulikevirus) enriched in CRC subjects [168], and when Coker et al. reported higher Malasseziomycetes and depleted Saccharomycetes and Pneumocystidomycetes in CRC patients [169].

CRC is a complex disease susceptible to a variety of diet and lifestyle factors, especially smoking, a well-known factor involved in the initiation and increasing the risk of CRC with a prolonged latency period [170]. Although the mechanisms of smoking-induced susceptibility to higher risk of CRC remain to be elucidated, preliminary evidence suggests a collective role of host, microbial, and smoking, such as intestinal and immune disruption, impaired clearance of pathogens, changes in the virulence of bacteria and fungi, and ingestion of bacteria that are present in cigarettes [171]. The fact as mentioned above that cigarette smoke or side-stream smoking decreases the amount of *Bifidobacterium* [124], mainly butyrate-producing bacteria with anti-inflammatory and anti-tumor molecule role [172] was highly consistent with the results that butyrate-producing bacteria are depleted in cancer patients [170]. In addition, in vitro and in vivo studies found that cigarette smoke not only decrease the fecal abundance of *Bifidobacterium* but also reduce its production of short chain fatty acids (SCFAs) [124, 173], immune-regulatory molecules modulating immune and inflammatory response within many diseases, and reductions in the concentration of SCFAs especially butyrate in colorectal tissues were demonstrated to be associated with the possibility of early stage CRC development [174]. Moreover, the smoking-related microbial changes may lead to altered epithelial mucin composition of the mucus layer and increased inflammatory response [175], which play pivotal role in the onset of CRC.

Stated thus, these studies suggested that alterations of gut microbiome may be an essential contributing factor to the initiation and development of this cancer in the context of smoking.

#### Systemic diseases

In addition to inflammatory bowel disease, dysbiosis of the gut microbiome has been implicated in many autoimmune diseases, including multiple sclerosis (MS) [176], rheumatoid arthritis (RA) [177], ankylosing spondylitis [178], systemic lupus erythematous (SLE) [179], and [180]. Human studies and mouse models support the role of the gut microbiome in predisposition to RA, such as reduction in bacteria belonging to the family Bifidobacterium and Bacteroides [181], and higher prevalence *Prevotella copri* [182]. Ye et al. also revealed higher abundance of *Bilophila* spp., and several opportunistic pathogens (e.g., *Parabacteroides* spp. and *Paraprevotella* spp.), together with a reduction in butyrate-producing bacteria *Clostridium* spp., and two genera of methanogens (*Methanoculleus* spp., *Methanomethylophilus* spp.) in BD patients [183]. MS has also been recently associated with changes of the intestinal microbiota with the study reporting depletion in species belonging to *Clostridium* and *Bacteroidetes* in Japanese patients with MS [184].

Smoking affects both innate and adaptive immune systems and plays dual roles in regulating immunity by either exacerbation of pathogenic immune responses or weakening of defensive immunity [185]. Smoking is a well-established risk factor for developing RA, SLE and MS [186–189]. Epidemiologic data showed positive association between the incidence of MS and smoking, the risk of MS increased with the number of pack-years of smoking increasing [190], smoking renders individuals with HLA–DRB1 shared epitope (SE) alleles susceptible to RA, stronger association existed in individuals carrying double copies of the SE [186]. The gut microbiome is responsible for maintaining homeostasis and function of host immune system indicating the probable essential role in changing the immune response that leads to autoimmune diseases like RA. As mentioned earlier, cigarette smoking could change the composition of intestinal microbiota [154], and some of the components play an important role in modulating intestinal homeostasis and immune responses to the enteric microbiota [155, 156]. The role of smoking involved in these diseases was assessed in both animal models and clinical trials, but so far no exact underlying mechanisms were identified. The gut microbiome may provide the missing link to this puzzle and help solve the mystery of the influence of smoking in autoimmune diseases, and this deserve the future research to further the understanding of the role of microbiome in systemic diseases.

## Conclusions

In conclusion, microbiome research has enormously developed in the last years tempting to move its steps to better characterize the human microbiome. Smoking is the risk factor of several diseases, it could impact human microbiome directly or indirectly through immunosuppression, oxygen deprivation, biofilm formation, or other potential mechanisms. None of the above mechanisms is well established, and adequate explanation of how smoking affects the microbiome is yet to be established. Microbiome has pivotal roles in the development of healthy immune responses, and oral, airway and gut microbial dysbiosis can contribute to local or systemic various diseases such as periodontitis, HIV infection, gastrointestinal cancer, asthma, COPD, CF, CD, UC, RA, MS. Evidence suggests the microbial dysbiosis in many diseases in smoky environment, but the causal relationship between microbiome alterations and disease progress remains enigmatic. More basic and clinical researches may help us gain more insight into the hugely complex net-work of smoking–microbiome–host interactions underlying the observed associations. Longitudinal studies integrating metagenomic, transcriptomic, metabolomic, methods with clinical results may help us ascertain the relationships between smoking, microbiome, and pathological mechanism in diseases.

## Data Availability

Not applicable.

## Abbreviations

COPD: chronic obstructive pulmonary diseases
CS: cigarette smoke
BALF: bronchoalveolar lavage fluid
CF: cystic fibrosis
CFTR: cystic fibrosis transmembrane conductance regulator
CD: Crohn’s disease
UC: ulcerative colitis
MS: multiple sclerosis
RA: rheumatoid arthritis
SLE: systemic lupus erythematous
